# The “Mellanby effect” in alcoholised e-scooter drivers

**DOI:** 10.1007/s00414-022-02920-z

**Published:** 2022-11-28

**Authors:** Katharina Zube, Michael Lau, Thomas Daldrup, Gina Maria Bruch, Anne Tank, Benno Hartung

**Affiliations:** 1 Institute of Legal Medicine, University Hospital Düsseldorf, Düsseldorf, Germany; 2 Institute of Mathematics, Heinrich Heine University, Düsseldorf, Germany; 3 Institute of Legal Medicine, Ludwig Maximilians University, Munich, Germany

**Keywords:** Mellanby effect, E-scooter, Alcohol, Resorption, Elimination, Matched pairs

## Abstract

**Purpose:**

Several studies tried to discuss and clarify the so-called Mellanby effect: Similar blood alcohol concentrations (BACs) supposedly lead to more signs of impairment in the phase of alcohol resorption than elimination. To assess this effect for alcoholised e-scooter driving, results of a real-driving fitness study were subanalysed.

**Methods:**

Sixteen subjects (9 females; 7 males) who completed runs at comparable BACs in the phases of alcohol resorption and elimination were chosen to assess a possible “Mellanby effect”. The data of the subjects was taken from a prior e-scooter study by Zube et al., which included 63 subjects in total.

**Results:**

In the phase of alcohol resorption, the relative driving performance was approx. 92% of the phase of elimination (*p* value 0.21).

Statistically significant more demerits were allocated to the obstacle “narrowing track” in the phase of resorption than elimination.

Subjects also needed significantly more time to pass the obstacles “narrowing track”, “driving in circles counterclockwise” and “thresholds” in the phase of resorption than elimination.

**Discussion:**

The most relevant obstacle to discriminate between the two different states of alcoholisation was the narrowing track. Insofar, measurements of the standard deviation of the lateral position (SDLP) might also be a sensitive component for the detection of central nervous driving impairment during shorter trips with an e-scooter.

Additionally, driving slower during the phase of alcohol resorption seems to be the attempt to compensate alcohol-related deficits.

**Conclusion:**

The results of the study suggest a slight Mellanby effect in e-scooter drivers.

## Introduction

A pecularity of alcohol (ethanol) is the comparatively stable relationship between dose and effects. With increasing blood alcohol concentrations (BACs), the magnitude of impairment increases [1, 2]. Nevertheless, with regard to road traffic, interindividual differences at similar BACs have repeatedly been described [3, 4].

Whether alcohol-related impairments are observable or not regularly depends on the complexity of the driving task [1]. In general, driving with a BAC of around 0.50 g/l increases the relative probability for single vehicle accidents by factor 2 [3]. Performance differences in comparable situations are often explainable by different states of alcohol habituation.

Besides habituation, several variables can directly or indirectly influence the driving-related effects of alcohol resp. the BAC. For example, the driver’s age is associated with different crash risks, which is likely linked to more increased alcohol-impaired crash avoidance skills in young drivers and risk-taking personality traits [5]. Similar BACs may provoke different levels of impairment at different times of the day as the circadian rhythm interferes [6, 7]. The ethanol elimination rate is usually (slightly) higher in women than in men [8, 9]. The state of alcohol hangover may also be associated with significantly impaired performances even if the BAC has returned to zero [10, 11].

A comparable BAC supposedly leads to more signs of impairment in the phase of alcohol resorption than in the phase of alcohol elimination. In other words, the BAC supposedly lags behind the alcohol effect. This observation is called “Mellanby effect”, since Sir Edward Mellanby was the first to describe it [12]. Mellanby carried out his experiments on dogs at the time, and he could therefore only assess gross motor abnormalities. The evidence for Mellanby effect is still under discussion, and driving safety skills might be more affected in the phase of alcohol elimination [13].

The presented work should clarify the question whether a Mellanby effect is evident in alcoholised e-scooter drivers. Results of a previously published study [14] were therefore re-analysed. As repeated e-scooter runs at different BACs were performed, several test persons completed the course with comparable BACs in the phase of alcohol resorption and alcohol elimination.

**Material and methods** (for details, see [14]).

### Test persons

Sixty-three healthy subjects, who were experienced in driving an e-scooter participated in the study (31 females, 32 males). Six subjects (3 females, 3 males) remained sober during the whole trial and served as control group. Two female test persons dropped out during the trial.

The test persons’ state of alcohol and drug soberness were checked at the beginning of each test day by breath-alcohol analyses and immunochemical screening of the urine.

Questionnaires were used to assess alcohol experience (AUDIT) and e-scooter experience.

For these re-analyses, matched pairs (e-scooter runs with comparable BACs with a maximum difference of 0.20 g/kg between the phase of alcohol resorption and elimination) were found in 16 alcohol-consuming test persons (9 females, 7 males) in the age range of 18 to 47 (median 24 years) (see below “Statistical analyses to evaluate a possible “Mellanby effect””).

### E-scooter runs and neurological examinations

Each of the 4 test days started at 10 a.m. and lasted until approx. 8 p.m.

The course was built on a non-public area (52 m × 18 m).

After accommodation to course and e-scooters (Tier, Modell ES 400B, Tier Mobility AG, Berlin), the sober run, which served as baseline, was performed.

Afterwards, alcohol consumption started (duration between approx. 2 h 22 min and 4 h 41 min). The amount of alcohol to be consumed was calculated in advance using the Widmark formula [15] in such a way that the BAC should reach a maximum value of approx. 1.30 g/kg.

Under the influence of alcohol, 3–4 runs were completed by each test person. All runs were videotaped for the purpose of later evaluation.

After each run, blood samples were taken.

### Course

**Fig. 1:**
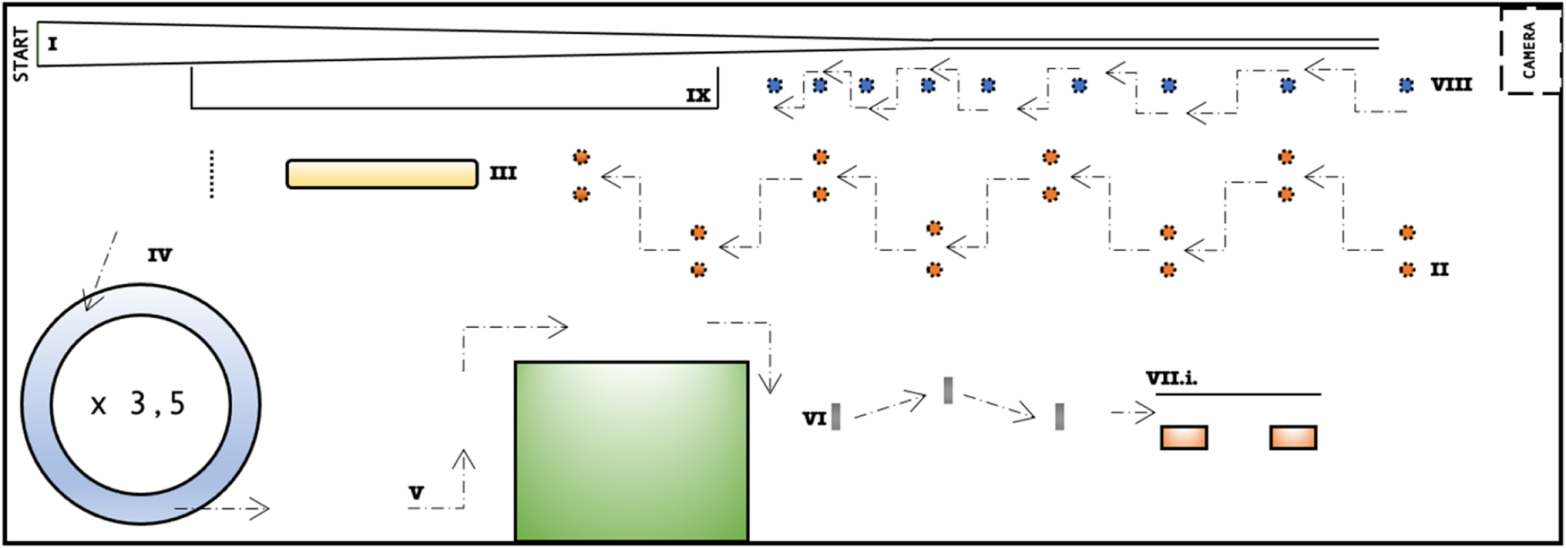
Course I: narrowing track; II: gate passage; III: gravel bed; IV: driving in circles counterclockwise; V: three turns with timely directional indication; VI: three thresholds; VII.i.: alley drive; VIII: slalom ride; IX: speed track) [14]

Nine obstacles had to be passed that can be seen in Fig. 1 (in chronologic order: narrowing track (45-m length); gate passage (spaced at 1.30 m); gravel bed of 6.90-m length; driving in circles counterclockwise 3.5 times; three turns with timely indication of the direction (left-right-left); three thresholds; alley drive (width: 1.05 m; length 5.55 m); slalom ride with decreased spacing (2 x 4 m; 2 x 3 m; 2 x 2 m; 1 x 1,5 m); speed track (17.7 m resp. 16.5 m)).

Two elements were slightly modified after the trial had already started: The speed track was shortened from 17.70 to 16.50 m, and a light signal was installed at the alley drive, which signaled the lane to be used. Furthermore, some subjects were asked to pass 6 of the abovementioned 9 elements a second time on each trip to better assess concentration losses or exhaustion effects during longer trips. Only the first passage was taken into account for further analyses.

A run through the 9 obstacles lasted on average about 1.5 min.

### Allocated demerits

Demerits were allocated for distinctive driving (coordinative or cognitive) features. Driving and medical features were evaluated separately.

More demerits were allocated for features that were considered more relevant to road traffic. For example, touching a boundary line (e.g. when driving straight ahead or during driving in circles) was allocated 1 demerit, crossing the line with one wheel was allocated 2 demerits and skipping a gate during gate passage was allocated 3 demerits.

### Statistical analyses to evaluate a possible “Mellanby effect”

BAC differences were set to a maximum of 0.20 g/kg between a test person’s run in the phase of alcohol resorption and alcohol elimination.

Persons were supposed to reach a maximum BAC of 1.30 g/kg. The alcohol to be consumed was calculated by the formula of Widmark [15].

To fulfill the demand of alcohol resorption, test persons were either still drinking alcohol or the termination of alcohol consumption was not longer than 60 min ago.

To fulfill the demand of alcohol elimination, test persons had finished alcohol consumption at least 90 min ago.

Basically, the added demerits per run were evaluated.

The added demerits per obstacle formed the “error score”.

The added demerits of all unchanged obstacles (I, II, III, IV, V, VI, VIII) formed the “absolute score”. If obstacles had to be passed twice, only the first passage of each obstacle during each run was included.

The “individual score” was calculated by comparing the results of the respective run under the influence of alcohol with the results of the sober run (with all test persons as control group). Subjects with an absolute score of zero points received one point to enable the calculations of all individual scores.

An “influence score” (*relative driving performance in different states of alcohol influence*) was determined by dividing the error score of the alcohol elimination phase by the error score of the phase of alcohol resorption.

The result of a statistical test was considered significant if the *p* value was less than 0.05.

## Results

Sixteen test persons (9 females; 7 males) aged from 18 to 47 years (median 24 years) were included in the analyses.

All 16 persons fulfilled the demand of alcohol resorption: Eleven test persons were still drinking alcohol, and 5 test persons had finished alcohol consumption no longer than 60 min before the run.

In the phase of alcohol elimination, the assessed 16 test persons had finished alcohol consumption between 90 and 187 min (median: 137 min) before the blood draw.

The average time interval between the run and the blood draw was approx. 9 min (range: approx. 1–15 min) in the phase of alcohol resorption and approx. 12:30 min (range: approx. 7–26 min) in the phase of alcohol elimination.

**Fig. 2:**
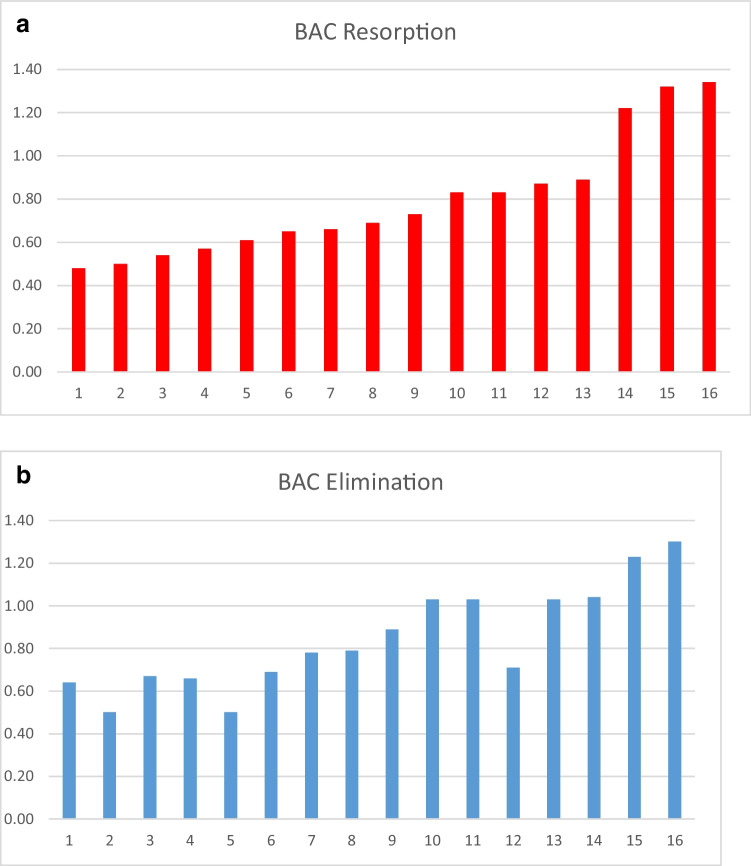
**a** Distribution of BAC of the 16 test persons in the phase of alcohol resorption (*x*-axis: test persons; *y*-axis: BAC in g/kg). **b** Distribution of BAC of the 16 test persons in the phase of alcohol elimination (*x*-axis: test persons; *y*-axis: BAC in g/kg)

The average BAC in the phase of alcohol resorption was 0.80 g/kg (Fig. 2a; median 0.71 g/kg; range 0.48–1.34 g/kg).

The average BAC in the phase of alcohol elimination was 0.84 g/kg (Fig. 2b; median 0.79 g/kg; range 0.50–1.30 g/kg).

Analyses of the sober control group revealed no hints for an improvement (e.g. due to habituation) or worsening (e.g. due to fatigue) during the trial [14].

**Fig. 3:**
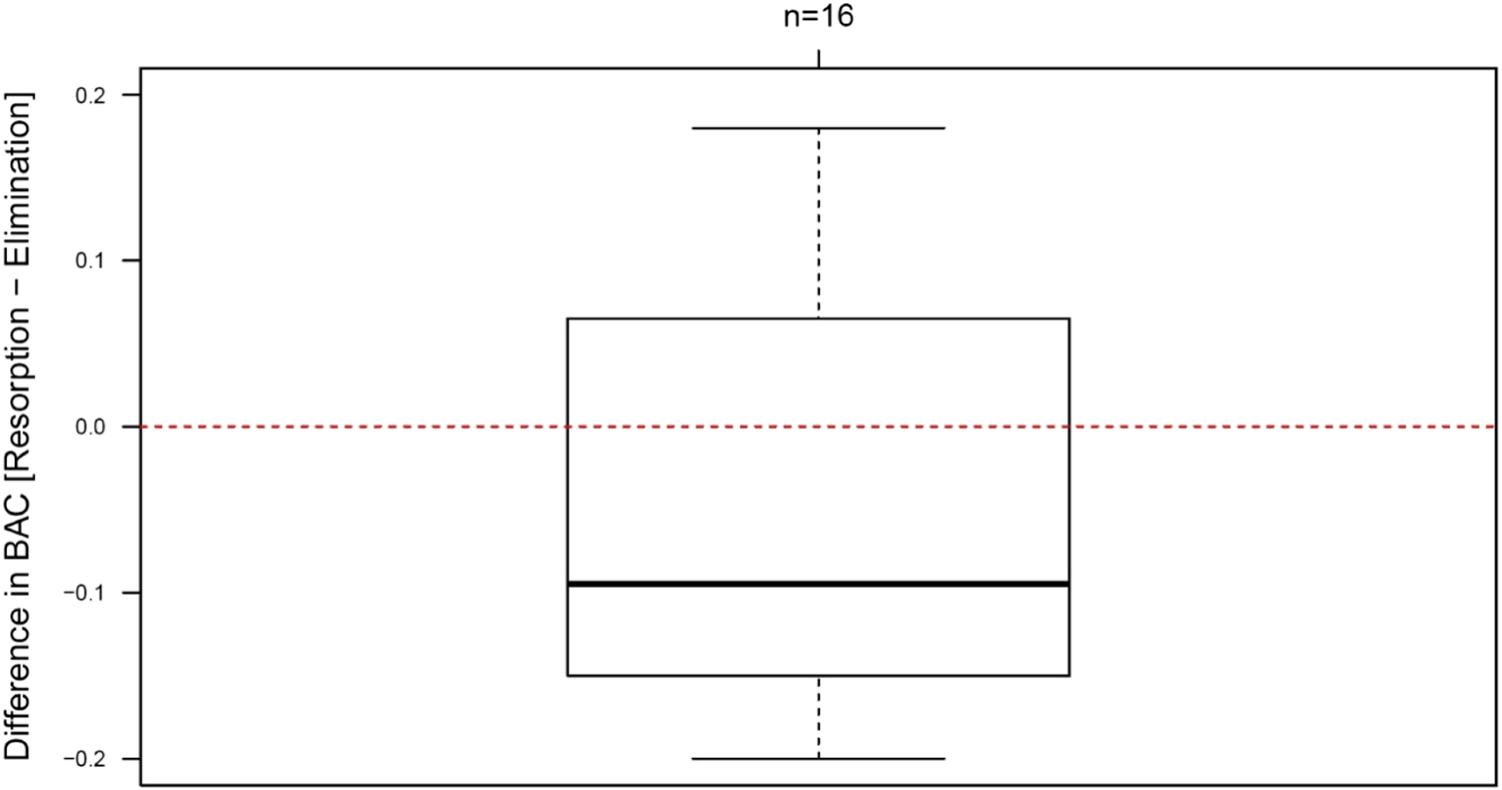
Median BAC difference between the runs in the phase of alcohol resorption and alcohol elimination. Results below 0 indicate a lower BAC in the phase of alcohol resorption. Box contains 50% of the tested persons, the line inside the box indicates the respective median, and the satellites indicate 25% of the tested persons.

Worse relative driving performances in the phase of alcohol resorption were seen despite of lower BACs in this phase (Fig. 3; median difference 0.1 g/kg).

**Fig. 4:**
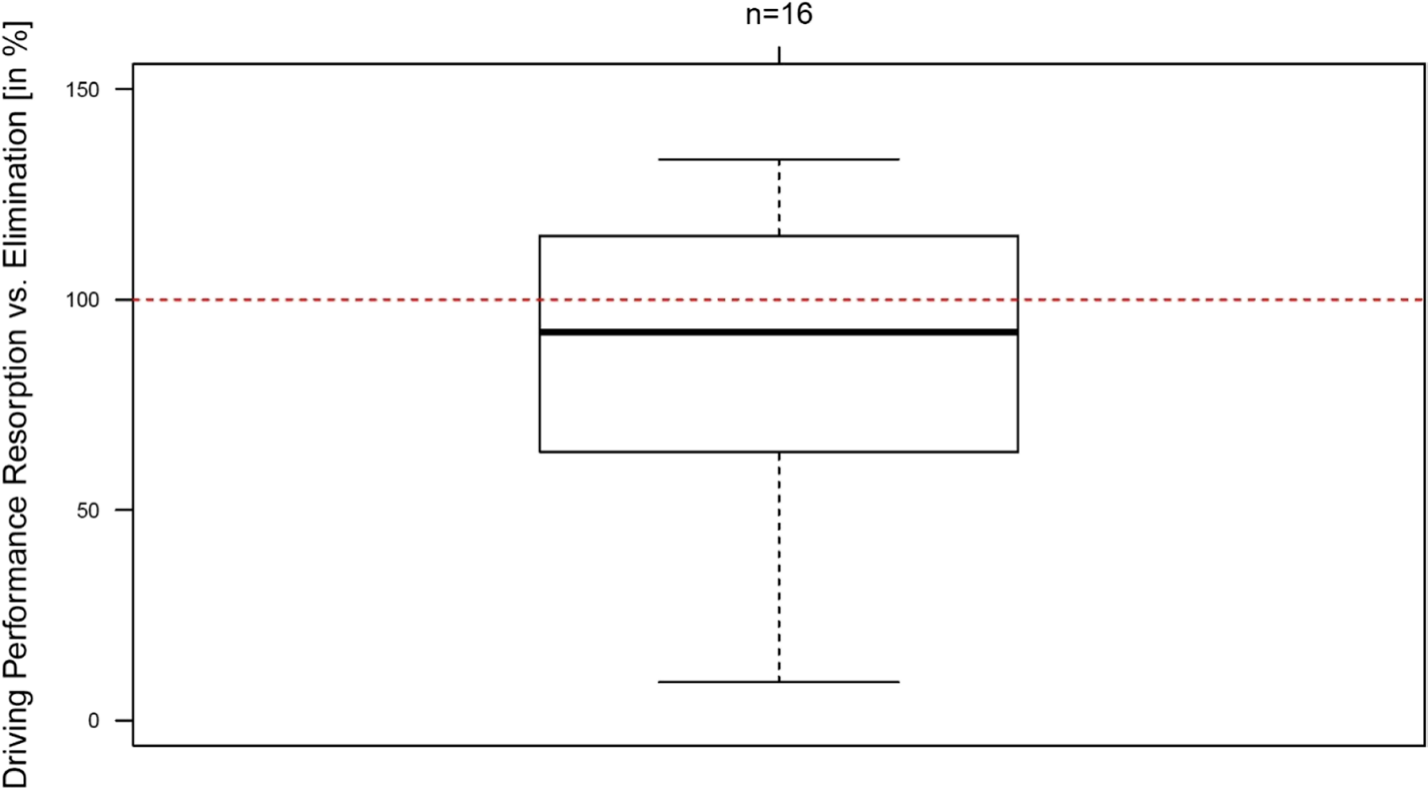
Relative driving performance in different states of alcoholisation (influence score). Results below 100% indicate a lower driving performance in the phase of alcohol resorption

Figure 4 shows the lower relative driving performance in the phase of alcohol resorption in comparison to the phase of alcohol elimination (*p* = 0.21; median 92.3%). This effect is independent from the BAC level (*p* = 0.24). The results were markedly influenced by a single test person (BAC resorption 0.65 g/kg, BAC elimination 0.69 g/kg), who received 3 demerits for his run during alcohol resorption and 8 demerits for his run during alcohol elimination.

Isolated evaluation for individual obstacles were carried out with regard to differences in allocated demerits and differences in times needed to pass the respective obstacle.

**Fig. 5:**
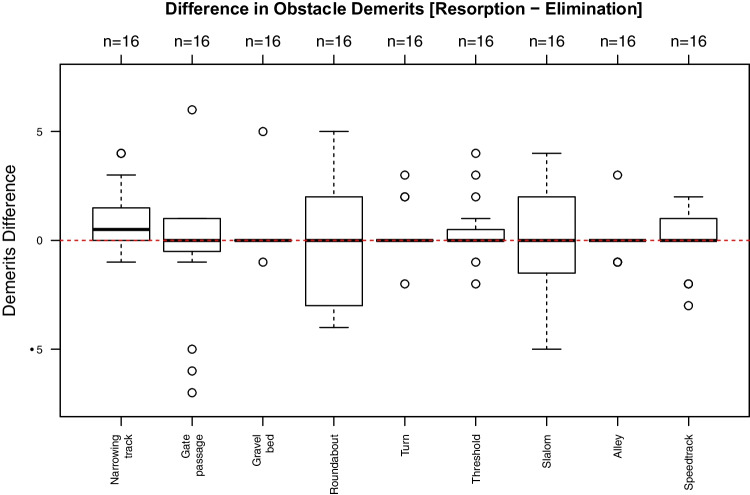
Differences (alcohol resorption vs. alcohol elimination) in allocated demerits (*y*-axis) for each obstacle (*x*-axis). Values above 0 indicate more allocated demerits in the phase of alcohol resorption. Only at the narrowing track the test persons had significantly more demerits in the resorption than elimination phase (*p* = 0.04) (gate passage: *p* = 0.43; gravel bed: *p* = 0.29; driving in circles counterclockwise (roundabout): *p* = 0.23; turns *p* = 0.23; thresholds: *p* = 0.15; slalom: *p* = 0.57; alley: *p* = 0.77; speedtrack: *p* = 0.45)

**Fig. 6:**
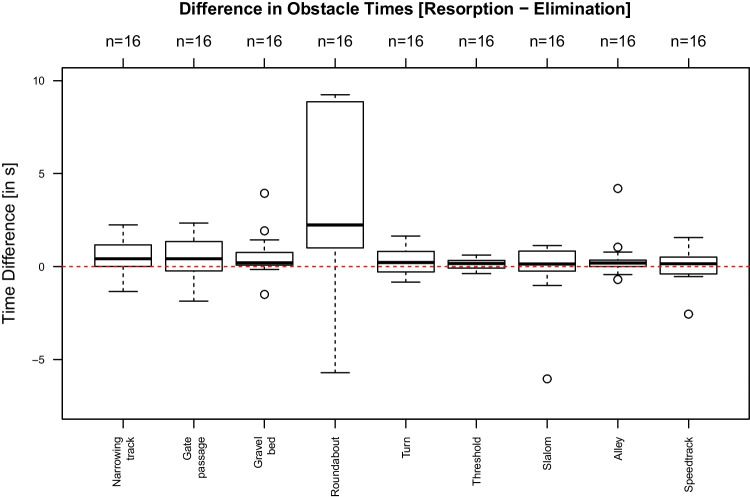
Differences (alcohol resorption vs. alcohol elimination) in the times needed to pass the obstacles in seconds (*y*-axis) for each obstacle (*x*-axis). Values above 0 indicate longer periods of time in the phase of alcohol resorption. At the obstacles narrowing track (*p* = 0.03), driving in circles counterclockwise (roundabout) (*p* =  < 0.01) and thresholds(*p* = 0.03) test persons needed significantly more time to pass the obstacle in the resorption than elimination phase (gate passage: *p* = 0.08; gravel bed: *p* = 0.05; turns *p* = 0.05; slalom: *p* = 0.59; alley: *p* = 0.07; speedtrack: *p* = 0.60)

Figures 5 and 6 show the of all allocated demerits for each obstacle and the differences in time needed to pass them. Allocated demerits were statistically significantly higher in the phase of alcohol resorption for the obstacle “narrowing track” (*p* = 0.04) only (Fig. 5). In addition, the times for passing the obstacles narrowing track (*p* = 0.03), “driving in circles counterclockwise” (“roundabout”) (*p* =  < 0.01) and “thresholds” (*p* = 0.03) were significantly increased (Fig. 6).

## Discussion

The Mellanby effect was examined under the contemplation of data from an e-scooter real driving test.

With a comparable BAC, there seems to be more impairment in the alcohol resorption phase than in the alcohol elimination phase. In their systematic review of this so-called Mellanby effect, Holland and Ferner concluded that test persons feel less drunk and are more willing to drive under the influence of alcohol when their BAC curve is on the declining branch. However, measures of traffic relevant skills seem to be worse in the elimination phase when BAC decreases [13].

The presented data indicate a possible Mellanby effect when driving an e-scooter. Interestingly, the most relevant obstacle to discriminate between the two different phases of alcohol consumption was the narrowing track. When considering all runs of all 63 test persons, performance on the narrowing track was significantly decreased from 1.01 g/kg [14]. This can be interpreted as a hint for the assumption that the standard deviation of the lateral position (SDLP) is also a sensitive component for the detection of central nervous driving impairment during shorter trips [16, 17].

When looking at the time needed to pass the obstacles, more obstacles were significantly conspicuous. When considering all runs again, an increased time to pass the obstacles narrowing track and driving in circles counterclockwise was significantly associated with more demerits in the course. The increased time to pass the thresholds in the state of alcohol resorption should be interpreted with caution as only few error points were obtained [14].

The presented subanalyses regarding time differences may be explained by the attempt to compensate self-recognized alcohol-related impairments.

Goldberg assumed a similar effect of alcohol on the motoric and sensor system, and psyche. He concluded that complex actions are appropriate to distinguish between the effects of alcohol resorption and elimination [18, 19]. The course which needed to be completed posed various challenges to the sense of balance, motoric skills and the need of high comprehension. The course can be considered as a complex task.

The presented data must be seen in the light of slightly higher BACs during elimination (median 0.1 g/kg), which likely weakened the Mellanby effect. As blood was drawn after each run, the BACs while driving must have been even lower during the phase of alcohol resorption, and even higher during the phase of alcohol elimination, which further weakens the effect.

From a toxicological point of view, it is easily understandable that fast BAC changes lead to more signs of failure than slow BAC changes: It was previously theorized that due to the strong blood flow to the brain, the central nervous effects of alcohol become apparent early [19, 20]. Our study design leaned towards a “realistic drinking event” (between 2:22 and 4:41 h to reach approx. 1.30 g/kg). Binge drinking with the most obvious central nervous effects was not examined [13, 19].

Holland and Ferner discussed common problems which make the studies of the Mellanby effect challenging: Most groups were rather small and consisted of young white men [13]. Our study included a greater range of ages and a vaguely similar proportion of women and men. Nevertheless, 16 subjects represent a rather small group, too.

Finally, we do not wish to ignore that the test persons drove under obvious test conditions. Already *Bschor* mentioned the problem of “spontaneous impulses” (sudden increase in concentration leads to a better performance) [19]. The subjects went through the course several times and were aware of completing the course as safely and fast as possible. So, the effect of spontaneous impulses cannot be excluded.

## Conclusion

First: The results of the presented study suggest a slight Mellanby effect in e-scooter drivers.

Second: Distinctive features may differ at similar BACs depending on the point of time the alcohol was consumed. In test situations, especially lower speed seems to be correlated to the phase of alcohol resorption. The termination of alcohol consumption should be taken into account for individual evaluation.

Third: E-scooter drivers could lull into a false sense of security by feeling safer at the descending limb and think using an e-scooter is a good alternative to a car or other motorized private transport.

Fourth: A study with a larger sample of subjects would be useful to finally assess the obtained hints.

## Data Availability

Anonymised raw data is available upon request (according to current scientific guidelines).
